# Thermotactic behaviour in lacustrine and riverine forms of *Salmo trutta* and its relevance to an emerging parasitic disease (PKD) in the wake of climate change

**DOI:** 10.1038/s41598-024-64137-x

**Published:** 2024-06-12

**Authors:** Albert Ros, Alexander Brinker

**Affiliations:** 1 Fisheries Research Station Baden-Württemberg, LAZBW, Argenweg 50/1, 88085 Langenargen, Germany; 2 University of Konstanz, Mainaustraße 252, 78464 Konstanz, Germany

**Keywords:** Lake trout, Brown trout, Cold-seeking behaviour, Temperature manipulation, *Tetracapsuloides bryosalmonae*, Global warming, Behavioural chill, Animal behaviour, Climate-change mitigation

## Abstract

The thermotactic response of brown trout (*Salmo trutta*) was examined with the goal to investigate potential effects of the emerging temperature-dependent fatal trout disease PKD (proliferative kidney disease). First the differences in cold-water preferences of two forms of brown trout, lacustrine (migratory) and riverine, were determined. Second, it was studied whether this preference was changed in fish infected with PKD. The experiment involved a one-week habituation period at 14 °C in a two-chamber runway followed by a week of 3 °C temperature difference between the two runways. The fish could freely move between lanes via an opening at the end where food was provided. The temperature manipulation was repeated twice, and there were 3 trials per experimental group. All fish developed a clear spatial preference in the test. Lacustrine trout demonstrated a preference for warmer water, while riverine trout preferred cooler water. This may increase the risk to PKD in the lacustrine form. Most strikingly, riverine trout experimentally exposed to *Tetracapsuloides bryosalmonae*, the parasite that causes PKD, demonstrated stronger cold-seeking behaviour than control fish. Cold seeking behaviour suggests the occurrence of a disease-induced behavioural chill response, which may play an important role in disease recovery. This demonstrates the significance of protecting river connectivity and cold-water sanctuaries as management strategies for preserving salmonid populations in a warming climate.

## Introduction

Europe, an important area for freshwater salmonids that prefer cool oxygen-rich waters^1–3^, is experiencing rapid warming^4,5^. This warming also affects the water temperature of mountain rivers^6,7^. To increase the resilience of salmonid populations to the impact of climate change, it is important to establish how these fish respond to changes in water temperature^8^. The brown trout *Salmo trutta* can be found in colder river segments when water warms in summer^9^, and it has been shown that Atlantic salmon (*Salmo salar*) use such cold-water refuges to maintain body temperatures lower than the mean river temperature^10^. Optimal temperatures differ among and within species due to factors such as food availability^5^and the timing of downstream migration. These factors can differ between lacustrine and riverine forms of brown trout, *i.e.,* those that migrate between a river and lake, *Salmo trutta f. lacustris,* and those that remain in rivers, *S. trutta f. fario*^11–14^. Water temperature can also impact salmonid disease incidence^15^. In particular, proliferative kidney disease (PKD) has been identified as an emerging disease^16^ in mountain streams in Europe^17^that poses a serious threat to wild^8,18–20^ as well as cultured^21,22^ salmonid populations. Therefore, the thermal response of a salmonid species may represent a balance of the benefits of warmer water with respect to migration strategies and feed abundance versus costs related to disease susceptibility.

In Europe, proliferative kidney disease (PKD) affects brown trout and rainbow trout (*Oncorhynchus mykiss*), and the causative parasite has also been identified in grayling (*Thymallus thymallus*), Atlantic salmon, and European whitefish (*Coregonus lavaretus*)^8^. It is primarily found in young-of-the-year, as fish surviving the disease become resistant^23,24^. PKD is caused by the myxozoan multicellular parasite *Tetracapsuloides bryosalmonae *^25–27^, which has a two-host life cycle^8,28^. Parasite spores released from the invertebrate host, freshwater bryozoans, enter salmonids via the gill and skin where they proliferate in the kidney and produce spores that in turn are infectious to bryozoans^28,29^. The parasite occurs mainly in the upper reaches of water bodies known as the trout region^19,30^. At water temperatures above 12 °C, parasites multiply in the kidney of their salmonid host^31,32^. When the water temperature exceeds 15 °C for more than 2 weeks, fish start exhibiting kidney damage caused by massive granulomatous infiltration and proliferation of the hematopoietic tissue, which in severe cases results in anaemia and high mortality^8,33–35^. Climate change is expected to increase this impact: streams that were formerly in the "PKD-safe" cool summer temperature range (< 15 °C) are increasingly exceeding the critical temperature^8,20,36^. Infected brown trout and rainbow trout maintained in cool water develop PKD resistance, which later allows survival in warmer parasite-infested waters^37–40^. Therefore, determining the effect of cool water refuges in river systems is critical to the conservation of trout populations.

Resilience to PKD might be critically depending on opportunities for salmonids to migrate and find river habitat that matches their optimal thermal conditions. For example, parasite infections are common in the Wutach River^30^, a near-natural river in southern Germany with relatively few barriers, good longitudinal and lateral connectivity, and natural reproduction of brown trout^41,42^. Although summer temperatures in many stretches in the Wutach exceed 15 °C for several weeks, the mortality rate of young-of-the-year brown trout is reported to be less than 15%^43^, and populations have declined only slightly in recent decades^30^. On the other hand, in more modified streams with similar temperature and PKD profiles, such as the Zollenreuter Aach, a far greater reduction in stock has been reported^40^. A possible explanation for such differences between trout populations is that unobstructed, near-natural rivers present a greater variety of microhabitats, including shaded areas and inflows of cool water from shaded tributaries or groundwater outlets. In such river systems, fish hypothetically have an increased opportunity to recover from parasitic infection by actively seeking out cooler segments of the river.

This study determined the behavioural responses of *S.* *trutta* to a relevant temperature gradient^44^. The main hypothesis was that negative thermotaxis in brown trout mitigates the clinical effects of PKD. The temperature differences tested were small but were expected to show an effect for multiple reasons: (1) the 15 °C threshold was not extensively exceeded in the mid-European river systems prior to recent climate change^7,36^; (2) it is the threshold at which PKD becomes virulent^6,30,35^; and (3) earlier studies detected a switch from positive to negative thermotaxis at a one degree gradient in the range of 15–16 °C in young of the year of *S. trutta*^44^*.* The setup of the study was also used to determine differences in temperature preferences between the lacustrine and riverine forms of *S. trutta* at this important temperature threshold. Such differences may result in life-history related differences in risk of PKD. The results have implications for understanding the impact of climate change on PKD in salmonids and for identifying the means by which this impact might be mitigated by river management.

## Materials and methods

### General methods

#### Animals and rearing conditions

In August 2021, 495 fingerlings of the riverine form of brown trout (*Salmo trutta f. fario*) and 165 fingerlings of the lacustrine form of brown trout (*Salmo trutta f. lacustris*) were purchased from local fish hatcheries. The riverine form was derived from a brown trout population in the river Kinzig on the western slope of the Black Forest (Rösch, Gengenbach, Germany), while the lacustrine form was derived from ancestors captured from Lake Constance (Staatliche Fischbrutanstalt, Nonnenhorn, Germany). Until the start of the trial, these fish were kept in tanks in the experimental facility of the Fisheries Research Station in a flow-through system with water taken directly from the adjacent Lake Constance at a depth of 30 m. The water temperature in the tanks was maintained at 14 °C with an oxygen content of ~ 12 mg L^−1^. The facility was illuminated using full-spectrum lamps with a lighting regime of 11:30 h/11:30 h light/dark, with a transitional phase of 30 min between phases (200 lx at the water surface). Fish were fed in the morning six days/week (INICIO Plus, Biomar Forellen Futter, Denmark). This schedule was chosen based on common practice in aquaculture securing and optimizing salmonid growth and welfare^45^. The feed and faeces were siphoned out at the end of the day.

#### Experimental design

**Figure 1:**
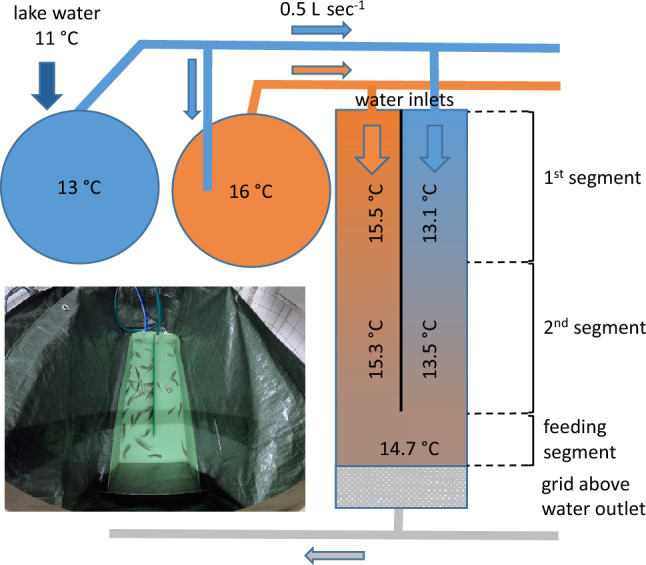
Experimental flow-through system: Water was heated in two tanks (circles). In the first tank, 1 L s^−1^ water was pumped from the lake. Half of the outflow of the first tank was directed toward the second tank. The outflow to the six runways was 0.5 L s^−1^. During habituation (week 1) and week 3 of the experiment, the water was maintained at 14 °C in both lanes. During week 2 and week 4 (experimental treatment), the water temperature was decreased to 13 °C (cold water) or increased to 16 °C (warm water). A 1-m high green curtain shielded each runway to prevent disturbance to the trout (see picture).

The setup comprised six replicate runways, each of which was 50 L (green fibreglass hatching tray, 17 × 40 × 120 cm; AGK Kronawitter GmbH, Germany). The first 90 cm of the runway was divided lengthwise into two lanes using an opaque Plexiglas^®^ sheet (Röhm GmbH, Germany), each with the water inlet at the start. A grid was placed to cover the water outlet. Between the grid and the lanes, a ~ 20 cm space was left where the fish were fed and where they could move freely between lanes (Fig. 1).

In Experiment 1, the temperature responses of 150 individuals of the riverine form were compared with those of 150 individuals of the lacustrine form of brown trout. In Experiment 2, the temperature responses of 150 brown trout (riverine form) exposed to *T. bryosalmonae* spores were compared with those of 150 unexposed control brown trout (riverine form). Each experiment was carried out in three replicate trials of 50 fish each. The trials in experiment 1 were initiated on 17.10.2021, and those in experiment 2 were initiated on 22.11.2021. Each trial continued for four weeks.

At the end of the trials, the fish were anaesthetized with clove oil *Caryophylli aetheroleum* (> 0.5 mL L^−1^) and killed by gill incision, after which the total length was measured to the nearest mm. At conclusion of experiment 1, the individuals of the lacustrine form were on average 0.7 cm larger than the individuals of the riverine form (riverine: 8.29 ± 1.04 cm, n = 144; lacustrine: 8.98 ± 1.01 cm, n = 141; *P* = 0.003), although the range of overlap was strong (6.0–11.8 cm). At conclusion of experiment 2 no significant difference was found in total length between the control and PKD-exposed group (6.3–14 cm; control: 9.83 ± 1.47, *n* = 140; PKD-exposed: 10.31 ± 1.43, *n* = 134; *P* = 0.15).

#### Temperature treatment during each trial

Water from two independently heated water systems (Red Line electric heater, Zodiac^®^, France) was supplied to the two lanes of the runway via an adjustable inlet (flow rate per inlet = 5 L min^−1^; Fig. 1). In the first week, the fish were allowed to acclimatize to the system for 7 days at 14 °C. On day 8, the temperature of the inflowing water in one lane was increased to 16 °C, and that in the other lane was reduced to 13 °C. On day 21, the temperature in both lanes was adjusted to 14 °C, and on day 28, the temperatures in the lanes were set to the opposite of those in the second week. The oxygen concentration at the end of the lanes ranged between 8.3 and 9.2 mg L^−1^. Heat exchange with the ambient temperature resulted in a temperature gradient in the lanes during temperature treatment (weeks 2 and 4): the temperature in the warm lane (inlet = 16 °C) was 15.5 ± 0.3 °C in the first segment and 15.3 ± 0.2 °C in the second segment; the temperature in the cold lane (inlet = 13 °C) was 13.1 ± 0.3 °C in the first segment and 13.5 ± 0.7 °C in the second segment. The water temperature in the feeding segment was 14.7 ± 0.5 °C. For analysis, the lanes were divided into two equal segments representing this gradient (Fig. 1).

#### Counting position of the fish in the runway

Recordings were made using six high-resolution GoPro HERO8 Black cameras (GoPro, Inc.) mounted two metres above the runways and programmed to take a photograph every 2 h during the light period. Fish in the photographs were counted using a machine learning algorithm written in Python and Google Colaboratory (Google, Inc.). The algorithm was trained with a set of photographs in which each fish was labelled against alternative objects such as food items. This provided labelled photographs that were individually checked and updated to obtain the final distribution of the fish across the runway lanes. In many cases, fish overlapped or clustered together, making it impossible to distinguish individuals, resulting in a total count lower than the number of individuals in the runway. On average, 87% of the fish were labelled.

### Experiment 2

#### Exposure of brown trout to PKD

Brown trout of the riverine form were exposed to *T. bryosalmonae* spores in preparation for Experiment 2, based on the method of Strepparava et al*.*^32^. On 30.09.2021, bryozoans (intermediate hosts) were collected from the Zollenreuter Aach (Ros et al*.* ^40^). Samples taken from these bryozoans yield spores matched 100% with *T. bryosalmonae*^30^. The day after collection, an infection suspension was prepared by mechanically grinding the bryozoans in lake water. Brown trout were divided over two keeping tanks, one PKD-exposure tank and one control tank. Before the infection suspension was added to the PKD-exposure tank (PKD-exposed group), the flow of water was stopped, the volume of water was reduced, and aeration was intensified. After 1 h, the flow was reactivated. The control tank was not exposed to the infection suspension.

#### Analysis of PKD

At the beginning and end of the experiment, a sample of brown trout from the PKD-exposed and control groups were tested for *T.* *bryosalmonae*, and kidney hyperplasia was scored on a scale of 0–5^33,38^. 25–55 µg sample of kidney tissue was homogenized (Bead Ruptor 4, OMNI International) and digested for 3 h at 55 °C (Mixer HC, Starlab), after which DNA was extracted using the PureLink^®^ Genomic DNA Mini Kit tissue kit (Invitrogen, Carlsbad, CA, USA) following the manufacturer’s instructions. The DNA was eluted in 75 μL of buffer and measured using a spectrophotometer (Nanodrop 2000c, ThermoFisher Scientific, USA), after which the concentration was adjusted to 300 ng µL^−1^ for quantitative polymerase chain reaction (qPCR) analysis. The method of Bettge et al*.*^31^was followed using the primers PKDtaqf1 and PKDtaqr1. A probe was applied with 6-carboxyfluorescein (FAM) as fluorescent reporter on the 5′ side and tetramethylrhodamine (TAMRA) as quencher at the 3′ side. Primers and probe were synthesized by Eurofins Genomics (Ebersberg, Germany). All analyses were carried out in duplicate using TaqMan master mix (TaqMan Universal Master Mix II, no UNG, Applied Biosystems, USA) on a QuantStudio 3 (Applied Biosystems, USA). A maximum of 32 cycles was used as threshold, as responses at higher cycles may result from unspecific amplification^31^.

### Ethics declaration

The study was carried out in compliance with the ARRIVE guidelines and permission for animal experimentation was granted by the Regierungspräsidium Tübingen, Referat Tierschutz (application: LAZ 03/21 G) according to the German Animal Welfare Act (TierSchG). Measuring the fish and taking probes for PCR was carried out after anaesthesia with clove oil *Caryophylli aetheroleum* (> 0.5 mL L^−1^) and killing the fish by gill incision.

### Data management and statistical analysis

For data management, Excel (2016 Microsoft Corporation, USA) was used. Binomial generalized linear modelling (GLM) was conducted to examine the treatment effects as indicated by the position of the individuals in the lanes (Fig. 1). To test preference for lanes in the habituation phase, the function “glm” of the “stats” package was used^46^. Binomial generalized mixed models (GLMMs) were used to examine the effect of water temperature treatment with trials as random effect using the function “bglmer” of the “blme” package^47^. The results of the analyses are summarized in Table 1. Body length differences and Euclidian distances between fish were tested using linear mixed models (lmer) with trial as random effect. Post hoc comparisons were carried out using the emmeans package^48^. Figures were drawn with Origin (OriginLab Corporation, 2022b; Northampton, MA, USA).

**Table 1:** Type III Wald *χ*^2^ tests of generalized linear mixed models (GLMM). Temperature effects are calculated using the preferred lane as reference (*bin* = binomial model).

	Parameter	Source of variation	*χ*^2^ (*df* = 1)	*P*
Experiment 1	*bin* (*n* in preferred lane, *n* rest runway)	Lake trout vs. brown trout (1)	31.551	< 0.001
		Cold vs. warm in preferred lane (2)	0.551	0.458
		Interaction (1) × (2)	6.783	0.009
	[in preferred lane] *bin* (*n* 1st, *n* 2nd segment)	Lake trout vs. brown trout (1)	33.748	< 0.001
		Cold vs. warm in preferred lane (2)	46.108	< 0.001
		Interaction (1) × (2)	92.832	< 0.001
Experiment 2	*bin* (*n* in preferred lane, *n* rest runway)	Control vs. PKD + (1)	0.201	0.654
		Cold vs. warm in preferred lane (2)	10.529	0.001
		Interaction (1) × (2)	2.918	0.088
	[in preferred lane] *bin* (*n* 1st, *n* 2nd segment)	Control vs. PKD + (1)	2.255	0.133
		Cold vs. warm in preferred lane (2)	10.813	0.001
		Interaction (1) × (2)	44.294	< 0.001

## Results

### Experiment 1: Differences between the lacustrine and riverine form

**Figure 2:**
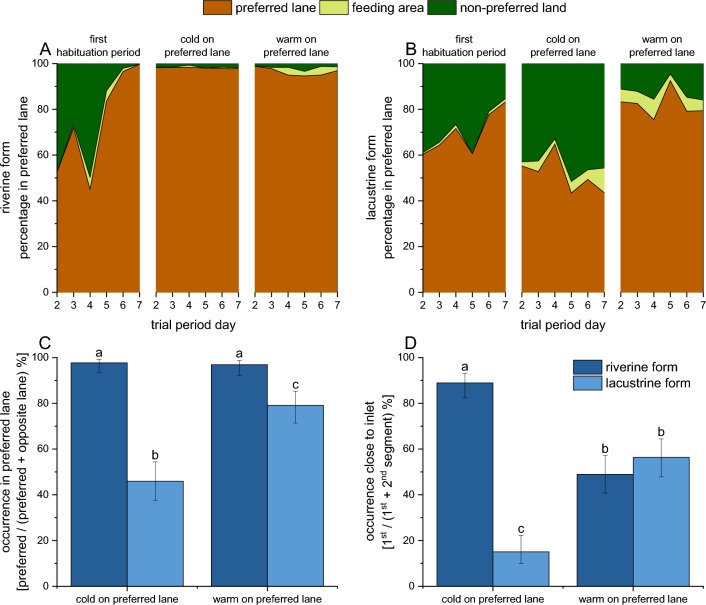
Experiment 1: Comparison of young-of-the-year of the riverine and lacustrine brown trout. (**A**,**B**) Changes in the relative distribution of the trout in the lanes over the 7 days of each trial condition. The preferred lane for each trial was the lane where most trout were counted at the end of the habituation period. (**C**,**D**) Relative distribution at the end of each temperature treatment relative to the preferred lane. In (**D**), the 1st segment of the lane is close to the inlet, the 2nd segment is close to the segment where the fish were fed and where the water of both lanes mixes. Columns with different letters are *P* < 0.05 in pairwise comparisons (Tukey’s HSD).

After the initial week of habituation at 14 °C, in all trials, the riverine form showed 100% preference for a specific lane (2 trials for the left lane, 1 trial for the right lane; Fig. 2A), whereas lane preference in the lacustrine form was 84% (2 trials for the left lane, 1 trial for the right lane; Fig. 2B). The GLM analysis showed this difference to be significant (*χ*^2^ = 31.3, *df* = 1, *P* < 0.001). Individuals of the riverine form were less dispersed across the lanes than individuals of the lacustrine form were (mean Euclidian distance of three closest fish at the conclusion of the habituation periods [*n* from two periods with some fish not able to position]: 4.0 ± 7.4 cm, *n* = 272 vs. 7.2 ± 10.3 cm, *n* = 270, *P* = 0.040).

Positions of the individuals during the trials were analysed relative to the average lane preference in each trial at the conclusion of the habituation week (Fig. 2C,D). During the temperature treatment (weeks 2 and 4), a significant difference between lacustrine and riverine form was found in the distribution across the lanes (*P* < 0.001), which was affected by temperature treatment (interaction temperature and form: *P* = 0.009; temperature: NS). Individuals of the lacustrine form tended to seek warmer water, whereas those of the riverine form remained in the preferred lane regardless of temperature treatment (Fig. 2C). Within the preferred lane, individuals of the riverine form moved away from the water inlet when the temperature was increased to 16 °C, and individuals of the lacustrine form moved away from the inlet when the temperature was decreased to 13 °C (form: *P* < 0.0001; interaction temperature and form: *P* < 0.001; temperature: *P* < 0.001) (Fig. 2D).

### Experiment 2: Effect of PKD on cold-water preference

Brown trout fingerlings from the breeder were not infected with *T. bryosalmonae* (qPCR CT > 32: 0 of 24 fish). At the start of the experiment 53 days post exposure, the PKD-exposed group had kidney parasite prevalence of 100% (n = 10, qPCR CT: 23.4 cycles), indicating mild hyperplasia on average (range 0–2; mean index = 1.2). Parasite DNA levels (qPCR) increased significantly over the four-week trial period (CT: 21.8 cycles, *P* < 0.001), and kidney hyperplasia manifested in all individuals (range 2–4; mean index = 2.9; *P* < 0.001).

**Figure 3:**
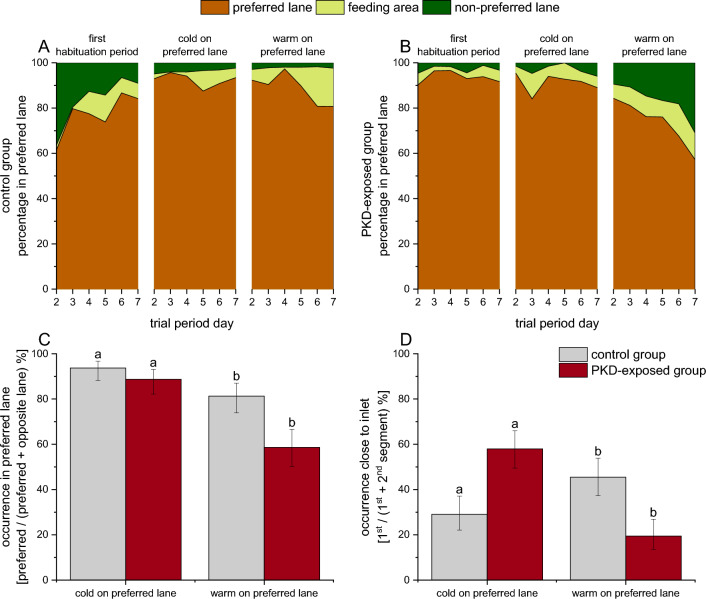
Experiment 2: Brown trout control and brown trout exposed to *Tetracapsuloides bryosalmonae* (PKD-exposed). (**A**,**B**) Changes in the relative distribution of the trout in the lanes over the 7 days of each part of the trial. 7 days of each trial condition. The preferred lane for each trial was the lane where most trout were counted at the end of the habituation period. (**C**,**D**) Relative distribution at the end of each temperature treatment relative to the preferred lane. In (**D**), the 1st segment of the lane is close to the inlet, the 2nd segment is close to the segment where the fish were fed and where the water of both lanes mixes. Columns with different letters are *P* < 0.05 in pairwise comparisons (Tukey’s HSD).

At conclusion of the habituation week, both the control and PKD-exposed group showed a strong lane preference (control: 1 left, 2 right, 90.4%; PKD: 2 left, 1 right, 96.5%; GLM: *P* = 0.99) (Fig. 3A,B). The PKD-exposed and control group did not differ significantly in dispersion across the lanes (mean euclidian distance of three closest fish at the end of the two habituation periods [week 1 and 3]; control: 6.6 ± 14.8 cm, *n* = 268; PKD-exposed: 7.2 ± 10.7 cm, *n* = 272; GLM: *P* = 0.29).

During the temperature treatment (weeks 2 and 4), the controls showed a significantly greater lane preference than did the PKD-exposed group (*P* < 0.001). Individuals in both groups were more likely to leave the preferred lane when the temperature was increased to 16 °C than when the temperature was decreased to 13 °C (*P* = 0.001; Fig. 3C). This temperature effect did not differ significantly between groups (interaction effect N.S.). However, within the preferred lane, there was a stronger tendency for PKD-exposed trout than for control trout to remain near the water inlet when it was flowing cold water and to move away when it was flowing warm water (Fig. 3D; PKD exposure: N.S.; temperature: *P* = 0.0010; interaction: χ^2^ = 44.29, *P* < 0.001).

## Discussion

This study investigated the thermotactic preferences of young-of-the-year *Salmo trutta* at 13 and 16 °C. Experiment 1 showed that the brown trout of the lacustrine form (*S. trutta f. lacustris*) tended to swim towards warmer water (positive thermotaxis), while brown trout of the riverine form (*S.* *trutta f. fario*) swam away from the warm water source (negative thermotaxis). In Experiment 2, exposure of brown trout to spores of *T.* *bryosalmonae* causing PKD increased the preference for cold water in comparison to that of the controls. The implications of these results are discussed in the context of climate change and the importance of connectivity and access to cold-water refuges in mountainous river systems.

### Thermotaxis in riverine and lacustrine form of brown trout

Both brown trout of the riverine and lacustrine form developed a strong preference for one of the two lanes of the runway during the first week of habituation, and this preference remained stable independent of water temperature treatment. This high site fidelity is in agreement with the results of a mark and recapture study showing that more than 80% of young-of-the-year brown trout remain in a river stretch over a period of 3 months^49^. However, the aggregation of fish into one lane in the habituation phase was unexpected, as the lanes had similar temperature and lighting conditions, and the feeding segment was common to both lanes. A possible explanation is that fish have a strong tendency to align swimming behaviour with their neighbours^50^, which is functional in the transmission of information among individuals about where to forage and when to flee predators^51^.

Lane preference was stronger in the riverine form than in the lacustrine form of brown trout. Relatedly, the riverine form showed a greater tendency to aggregate than did the lacustrine form. This difference is unlikely to be an effect of differences in inter individual competition between forms, as research has not indicated differences in individual aggression between lacustrine and riverine forms of *Salmo trutta *^52^. A greater tendency to disperse in the lacustrine form might be related to the pre-migration smolting process that ultimately results in brown trout of the lacustrine form migrating downstream from the river to the lake^53^. In contrast, the greater lane preference of brown trout in the riverine form might reflect site fidelity leading to low dispersal and residency in rivers^54^.

In the current study we show that different forms of the brown trout significantly differ in their thermotaxic response to a temperature increase of 13 to 16 °C. Within a lane, the riverine form moved away from the inlet when it was switched to warm water. The lacustrine form exhibited positive thermotaxis in swimming away from the cold-water lane and toward the warm water source. Previous research has shown that at this range of temperatures (13–17 °C), young of the year of brown trout switch from positive to negative thermotaxis^44^*.* Furthermore, although temperature preferences appeared to increase with increasing acclimation temperatures, these preferences were found to be lower than the acclimated temperature when water temperature increased above 15 °C^55^. A possible explanation for the current result is therefore that the riverine form has a low threshold to switch from positive/neutral thermotaxis to negative thermotaxis, and that this threshold was not reached at 16 °C yet for the lacustrine form. Also behaviour in response to temperature changes differed between forms. The riverine form moved less frequently between warm and cold lanes than did the lacustrine form. Brown trout of the riverine form are known to migrate little within a river in the first half year of life and are often observed to move in an upstream direction^54^. Positive thermotaxis in lacustrine form could induce migration downstream into warmer segments of the river, hastening maturation and triggering migration (smolting)^56–58^. The current results show that this behaviour occurs at 15 °C, which is a critical temperature for PKD as both *T.* *bryosalmonae* spore load and the chance of clinical disease due to infections increase at this temperature threshold^8^. Therefore, juveniles of the lacustrine form may be particularly susceptible to PKD compared to the riverine form during their migration from hatching in headwaters to the lake where they will spend much of their maturation. Successful migration might be critically dependent on availability of cold-water refuges and shade, not only at the headwaters but all along the migratory route through connecting rivers.

### The effect of PKD on thermotaxis

Brown trout of the riverine form, experimentally treated with spores of the parasite *T. bryosalmonae*, showed mild kidney hyperplasia indicative of PKD^59,60^ as well as significantly lower thermotaxis than unexposed controls. In the currently studied system, this is of interest to both the host and parasite: severe kidney damage through PKD reduces the survival of the salmonid host^35,43^, and destruction of nephrons in diseased kidneys limits mature spores leaving the kidney via urine^61–63^. Thus, both host-induced and parasite-induced mechanisms may explain the observed cold-seeking behaviour in *Salmo trutta* in response to PKD. Behaviour manipulation has been described as a mechanism that increases between-host parasite transmission^64^. However, in myxozoan diseases that cause manipulation of host behaviour, this effect has been attributed to central nerve damage^65–67^, whereas *T.* *bryosalmonae* is not found in brain tissue but in interstitial tissue of the kidney.

Disease-induced thermotaxis is a common thermoregulatory response to bacterial infections in fishes, much like fever in mammals^68^. Such “behavioural fever” responses are regulated by the hypothalamus via inflammatory cytokine release^68,69^. Cold-seeking behaviour in response to infection, known as “behavioural chill”, has rarely been reported^70,71^. In fish it has been described for sticklebacks *Gasterosteus aculeatus* in response to later stages of infections with *Schistocephalus solidus*^72^, and has been reported in broad-nose pipefish *Syngnathus typhle* in response to experimental infection with *Vibrio*^73^. In these cases, as in the present study, chill behaviour might be beneficial for mounting an immune response to infection, as the growth rates and virulence of the respective parasitic organisms decrease with decreasing temperature. Cold and warm water seeking behaviour depending on water temperature has been described for brown trout infected with glochidia of the pearl mussel *Margaritifera margaritifera*, although it was proposed that this might be behavioural manipulation by the parasitic organism^74^.

As metabolic processes accelerate with increasing temperature^75^, the virulence of *T. bryosalmonae* is likely to increase at higher temperatures^8^. A study of *T. bryosalmonae* infections revealed differences in immune function and parasite intensity in fish kept at 12 vs. 15 °C^76^. When the temperature remains at > 15 °C for several weeks, infection may result in damage to hemopoietic tissue^77^and significant mortality^35,43^. Providing cool water (< 15 °C) has been shown to be the most effective remedy for *T. bryosalonae* infections in culture facilities^38,78^. In such cool water, brown trout tolerate kidney infection caused by *T. bryosalmonae *^32^ and eventually regenerate renal tissue^61,79,80^. The current results indicate that brown trout infected with PKD actively seek colder water, which might effectively function as a “behavioural chill” to fight-off the disease.

### Thermotaxis and river management

European salmonid populations are declining alarmingly, and this has been related to the warming of rivers through climate change and emerging PKD^19,20,30,81^ in addition to factors such as food availability, predation, human water use, and physical barriers^8,82–84^. The results of the present study highlight the importance of free-flowing waterways^85^ for allowing salmonid access to river stretches that correspond to their temperature preferences^5,9,86,87^ and for seeking cold water in response to disease (PKD). Even adults, which are less prone to develop PKD^8^, have been found to prefer colder water when temperatures rise to temperatures above 20 °C^55^. Providing free corridors by means of fish passes or dam openings and preserving cold water refuges (ground water, shaded areas, cold water tributaries)^88,89^ must be prioritized in the conservation of salmonid river ecosystems. While river temperatures are likely to increase with ongoing climate change^6,90^, cold water refuges may buy urgently needed time for salmonids to adapt to local climate conditions^58,60,91^. Cold-seeking behaviour is currently being investigated in wild populations of brown trout that are affected by PKD.

## Conclusions

The lacustrine form of brown trout exhibit positive thermotaxis, which guides lakeward migration but also increases exposure to PKD, while the riverine form of brown trout exhibit negative thermotaxis, together with strong site preference, which is likely to lead to riverine residence. PKD-induced negative thermotaxis in brown trout is a rarely described “behavioural chill” response that likely has healing effects and thus highlights the importance of cold refuges during periods of hot weather.

### Supplementary Information

Supplementary Information.

## Data Availability

The data that support the findings of this study are openly available in figshare at https://figshare.com/s/876846084a0f985eccef.
